# Financial big data management and intelligence based on computer intelligent algorithm

**DOI:** 10.1038/s41598-024-59244-8

**Published:** 2024-04-24

**Authors:** Jia Liu, Shuai Fu

**Affiliations:** 1 School of Economics and Management, Harbin University, Harbin, 150086 Heilongjiang China; 2 Changchun Humanities and Sciences College, Changchun, 130117 Jilin China

**Keywords:** Financial big data, Big data management and control, Intelligent analysis of management and control, Intelligent computer algorithms, Environmental sciences, Environmental social sciences

## Abstract

With the acceleration of China’s economic integration process, enterprises have gained greater advantages in the fierce market competition, and gradually formed the trend of grouping and large-scale. However, as the scale of the company increases, the establishment of a branch also causes many problems. For example, in order to obtain more benefits, the business performance of the company can generate false growth, resulting in financial and operational risks. This paper analyzed the current situation and needs of enterprise financial control from two aspects of theory and practice, combined with specific engineering projects, taking ZH Group as an example, according to the actual situation of the enterprise. The article first introduces the basic situation of the enterprise; Then, the financial control strategy was designed, and different modules were designed to achieve financial control; Afterwards, use a reverse neural network to evaluate the effectiveness of financial management and risk warning; Relying on particle swarm optimization algorithm to seek the optimal solution and applying it to financial management and risk warning, in order to improve the level of introspection and risk management in decision-making. Finally, the value of computer intelligence algorithms in financial big data management is evaluated by constructing a financial risk indicator system. Through the analysis of enterprise financial management, the total asset turnover rate of ZH Group decreased by 0.39 times in 5 years. After 5 years of adjustment of the company’s business, the company’s overall operational capabilities still needed to be improved, and the company’s comprehensive business capabilities also still needed to be improved. Therefore, the application of intelligent algorithms for financial control is very necessary.

## Introduction

With the continuous deepening of the process of enterprise informatization, the traditional financial control model can no longer meet the high-quality and high-efficiency requirements of large enterprises for financial control, and private enterprises such as ZTE and Huawei have also begun to explore financial management^1,2^. Traditional financial management and control methods are difficult to cope with large-scale and highly complex data, and their financial management methods are difficult to monitor and warn of potential risks and problems in real time^3^. This paper chooses ZH Group, a large-scale enterprise in many fields, as the research object, covering many different fields, among which construction is the main one, supplemented by various business forms such as service industry and commerce. These enterprises have many operating scales and levels, and the complexity of business management is also high. Therefore, the financial management and control of enterprise groups is relatively difficult, and in the past, a decentralized financial control model was basically adopted^4^. Compared with other industries, the group control of a multi-industry group is highly centralized and decentralized, and data sources are difficult to obtain, which makes the group's financial control relatively weak. Meanwhile, the available system tools must have integrated, standardized, and definable functions, as well as the flexibility to adapt to the rapidly changing policies of the enterprise. At present, the popular computer intelligent algorithm is more in line with the current actual needs because of its advantages of being able to quickly process a large amount of data, dynamically adjust according to different situations, and making decisions based on objective data and models^5^. In financial management, computer intelligent algorithm can improve the efficiency and accuracy of financial management, reduce human errors and omissions, and at the same time, it can realize automatic decision-making and forecasting, providing strong support for financial management^6^. Therefore, this article will study the financial big data management and intelligence of computer intelligent algorithms, hoping to provide more methods for financial data management and better achieve the intelligence of financial management through this research.

The use of intelligent algorithms to analyze the financial management and control of enterprises is one of the hot topics of the moment. Intelligent algorithms help enterprises improve financial management efficiency and reduce the risk of human error and fraud. Intelligent algorithms can also analyze big data, predict market trends, and provide more targeted financial strategies for enterprises. The application of intelligent algorithms in financial management can monitor the financial status of enterprises in real time, identify potential problems in a timely manner, and ensure the stable operation of enterprises. Among them, Li analyzed the job of monetary specialist organizations in surveying SME store network credit and how they can assist SMEs with acquiring store network supporting through computerized stages utilizing enormous information examination^7^. Lăzăroiu states that machine learning algorithms can streamline the payment operations capabilities and the timeliness of the process, ensure the smooth operation process, assess the risk, and detect fraud and money laundering through historical data and customer behavior instantly payment network and infrastructure analysis^8^. Nguyen uses a multidimensional descriptive analysis to familiarize people with the role of Big Data, Artificial Intelligence, and Machine Learning technologies in the financial technology roadmap and stated that AI and machine learning technologies are relevant to the future challenges of AI ethics, regulatory techniques and smart data utilization^9^. To better understand the challenges and applications of AI in the delivery of financial services, Mogaji proposed a conceptual framework for AI related to financial services marketing that captures and emphasizes the interactions between customers, banks and external stakeholders, and regulators, and the study provides empirical insights into the opportunities, prospects, and challenges of the use of AI in financial services marketing^10^. Iurasov A’s based on the mathematical modeling of retail merchandise assets and the calculation of combined profit margins, he proposed a method for retail merchandise asset optimization^11^. The research of these scholars helps enrich financial management and control methods, providing more ideas for enterprise financial management. However, due to insufficient data sources and insufficient understanding of the enterprise framework in their research, their research is still in the theoretical stage and there is no substantial evidence to prove the feasibility of the ideas. This leads to unclear application effects of intelligent algorithms in financial management and control, More evidence is needed to prove it.

Using big data and artificial intelligence algorithms to analyze corporate financial management is a very innovative idea. Big data and artificial intelligence algorithms can deeply mine company financial data, reveal hidden correlations and trends, and provide strategic insights. These advanced technologies can process large-scale data, predict financial risks, and assist enterprise decision-makers in making wise financial decisions. Among them, Alnsour planned to concentrate on the impact of Saudi Arabia's financial control decision and examined measurable contrasts of exploration factors against several hierarchical variables^12^. The scientific achievement of Kosova’s research was to refine the definition of national and regional budget guarantees, and to confirm the conceptual model of financial control under the condition of decentralization^13^. Jianu recommended that monetary control, as a piece of inner administration and financial control, is likewise an action for knowable service elements that is significant for the proficient, successful and practical utilization of the assets dispensed to them^14^. Bharadiya argues that financial intelligence aims to improve the timeliness and quality of data to enable managers to better understand their company’s position among competitors, and he attempts to provide a framework for the development of enterprise intelligence systems that combine operational and historical data with analytical tools to provide financial managers and decision makers with important competitive information^15^. Padmanaban H delves into the the challenges and opportunities inherent in utilizing reference data in artificial intelligence for comprehensive financial data analysis, and he points out that there are opportunities for innovation within these challenges, including advanced data analytics, artificial intelligence, and blockchain technologies that have the potential to improve the accuracy, efficiency, and transparency of financial data analysis^16^. Scholars’ exploration of big data and artificial intelligence algorithms in financial management can provide a certain theoretical basis for this study, which is conducive to promoting the transformation of financial management towards intelligence. However, due to the limitations of traditional framework thinking and definitions in scholars’ research, it is difficult for their research results to be highly integrated with intelligent algorithms and fully leverage their advantages, resulting in a lack of significant improvement in financial management efficiency, and the accuracy of financial prediction models has not been improved. The research results do not have much reference value.

In order to better achieve financial big data management and intelligence, this article combines computer intelligence algorithms, conducts in-depth research on enterprises, and introduces BP neural network and particle swarm optimization algorithm to achieve financial big data management and intelligence. Empirical research has found that the financial prediction model constructed in this article has better accuracy. The innovation of this article is reflected in the following aspects: computer intelligence algorithms effectively integrate and process financial big data, break the limitations of traditional methods, and provide a solid foundation for data-driven decision-making. Intelligent algorithms and other technologies automatically mine the inherent patterns of data, improving the accuracy and real-time performance of financial forecasting and risk assessment. This article studies and improves the theoretical framework of financial big data management, providing direction for future research and promoting continuous innovation in this field. Interdisciplinary integration and transparent design ensure the fairness and rationality of the decision-making process, providing support for the intelligent transformation of the financial industry.

## Enterprise financial control and intelligence

### Introduction to the current situation of the enterprise

**Figure 1:**
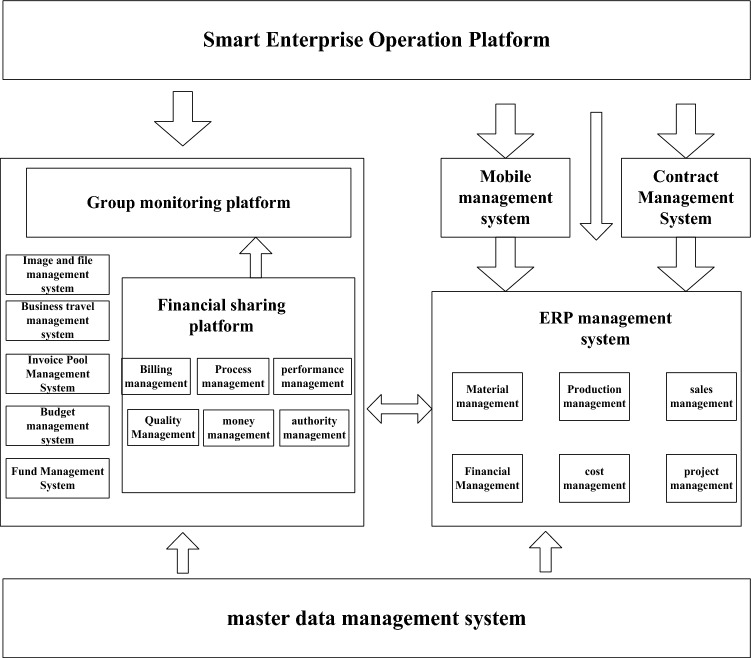
Status quo of enterprise informatization and financial control integration.

This paper takes ZH Group as the research object. ZH Group is a large-scale construction engineering group, and its financial information management level has been improved year by year. ZH Group has 21 secondary management organizations and more than 600 management organizations at all levels, with different business models and products, different management models, management styles, organizational structures, and different management priorities and management methods^17,18^. This paper makes a detailed description of the enterprise’s internal control from the perspectives of the current situation of informatization and the current situation of financial control, and based on this, it describes the informatization financial control system. The specific content is shown in Fig. 1.

As can be seen from Fig. 1, ZH’s Enterprise Resource Planning (ERP) system is now fully established. The enterprise resource planning system is mainly divided into four parts. Firstly, it is an enterprise intelligent operation platform, followed by an organizational supervision platform, which mainly includes a financial sharing platform; Next is the ERP management system, which includes 6 modules, and finally the master data management system. Because the business scope of the group's internal enterprises is extensive and complicated, the design and implementation of ERP are based on the actual operation and management of each unit. A standard construction guide and specification has been formulated, led by each second-level unit, and centralized deployment in the second-level unit. Combined with the business needs of each legal entity, module selection and implementation are carried out on the standard framework. The ERP system aims to improve the integration of business and finance of individual companies, and according to different operating conditions, improve the integration of business and finance of enterprises, and reduce the amount of manual financial accounting^19,20^. The group has integrated the accounting system to ensure that the accounting statements of each department are consistent. According to the business management process of the enterprise, it has formulated a standardized process from procurement to payment, sales to collection, production to cost. It also sets up the project promotion team of the ERP Group to support the project construction of various secondary enterprises and ensure the implementation of the standards. In the process of promotion and optimization of ERP projects, there are still unsynchronized launch, different solutions, different products, difficulty in post-optimization, and integration with external systems, as well as many difficulties caused by non-standardization, which affect the overall informatization efficiency of the enterprise.

### Overall strategy design of group financial management and control

**Figure 2:**
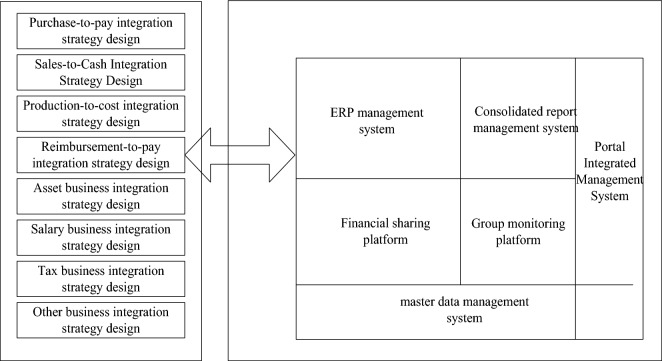
Design diagram of the overall strategy of the group’s financial management and control.

This chapter takes the group financial control as the core, the information system architecture as the foundation, and the information technology as the foundation. Through the integration of technology and management, breakthroughs have been made in theory and practice. From the perspective of financial management and information system implementation, the overall idea and implementation route are designed, and the overall structure of the entire design is illustrated in the form of graphics, as shown in Fig. 2.

As can be seen from Fig. 2, ZH Group is a large-scale and diversified group. It is difficult for its headquarters to have enough resources to control its subsidiary companies. The financial strategy design of the group is mainly divided into 8 parts, namely procurement and payment integration strategy design, sales and cash integration strategy design, production cost integration strategy design, repayment and payment integration strategy design, asset business integration strategy design, salary business integration strategy design, tax business integration strategy design, and other business integration strategy design. These strategic designs are ultimately integrated into the enterprise resource planning system.It maintains the value of assets through investment, finance and other means. Therefore, financial control is the first choice for enterprise management, and in such a large-scale enterprise, without the support of information technology, it is impossible to realize the financial control of the enterprise^21^. When considering the design of the group's overall financial control strategy, people must never leave the perspective of financial management, let alone the information system to design financial control, and the financial control of the group is to meet the business needs of the enterprise by means of information technology.

1. Strategy design of sales-to-collection integration

Sales is an important business activity, which includes sales plans, sales areas, sales customers, etc., and conducts comprehensive management and statistics on sales volume, sales amount, profit, performance, customer service, etc.^22^. According to customer data classification, effective management of customer data enables customers to better provide customers with personalized services and improve customer satisfaction. In the sales confirmation stage, including the signing of the sales contract, according to the terms of the contract, it can include prepayment for sales, confirmation of receipt, issuance of sales invoices, and billing and collection. Even if there is no invoice or funding problem, once the ownership of the goods has been transferred, the clearance must be carried out to ensure the accuracy of the clearance. From sales to receipt, the relationship between enterprises and finance can be seen. By reducing, reorganizing, refining, and standardizing the commonality of the entire business process, the overall operational efficiency of the enterprise can be improved.

(2) Strategy design of production-to-cost integration

Production management is the central link of enterprise resource management, which organically combines all the production processes of the enterprise, and combines the procurement management in the material management with the back-end sales management, so that the inventory management of the enterprise is more efficient. At the same time, the original scattered production processes are connected in series, so that the production process of products can flow automatically, so as to achieve continuous production and early warning of product and delivery requirements. Production management is guided by the production plan. According to the pre-established production plan, it is decomposed into executable and standardized work tasks and completed by each production department. During the entire production process, when the entire production process is completed, an enterprise’s inventory can be formed. From the whole process of production and operation to financial expenses, the connection between enterprises and finance can be seen. According to the commonality of the overall business process of the enterprise, the process is tailored, and the process is reorganized. The process tasks are also refined and standardized, so as to improve the overall operation and management efficiency of the enterprise.

(3) Strategy design of other business integration

In addition to some of the more concentrated businesses mentioned, there are many other businesses, which are generally classified as the general ledger of the company^23,24^. For ZH Group, other businesses based on financial management include fund management, financial investment, special general ledger, financial adjustment, statement management, standard attachment management, etc. In the fund management business, fund settlement runs through all links such as procurement, sales, reimbursement, etc. There is no separate design, and the centralized management of funds included is also carried out within the enterprise.

### Realization of group financial control strategy

In the design of the corporate financial control strategy, the design idea of the corporate financial control strategy is expounded in detail, and the corporate design concept is transformed into a concrete implementation, which is linked with the actual management problems of the enterprise. Through the analysis of ZH Group's strategic design, this paper examines its strategic implementation from the perspective of business logic and system logic, and gives some specific application examples. The implementation of the overall strategy mainly starts from the perspective of financial management and the implementation of the information system, and is classified according to the overall strategy implementation.

1. Realization of integrated sales-to-collection strategy

**Figure 3:**
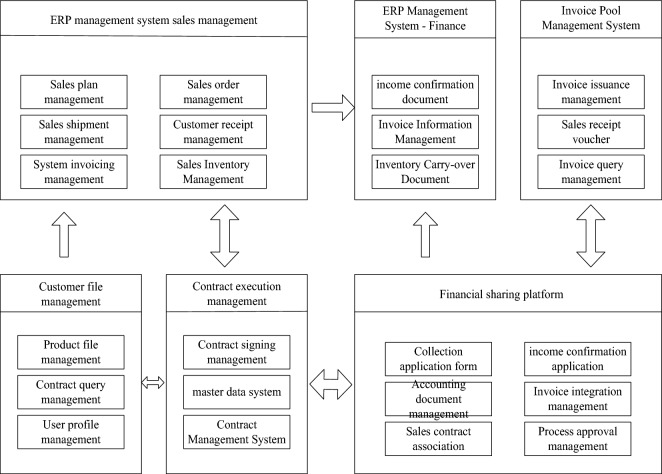
Integration diagram for the implementation of the sales-to-cash integration strategy.

In terms of the integration of sales and collection, it is necessary to clearly divide the ERP system, the financial sharing platform, the invoice pool management system, the contract management system and the master data management system, thereby realizing an integrated application from sales to collection. In the comprehensive application, it is necessary to give full play to the functional advantages of each system, and do a good job in the integration management between each system, so as to achieve a better display effect after implementation, as shown in Fig. 3.

The sales to cash integration strategy in Fig. 3 is divided into 6 modules. The first module is sales management in the ERP management system, the second module is finance in the ERP management system, and the third module is the invoice pool management system; The fourth module is customer file management; The fifth module is contract execution management; The sixth module is the financial sharing platform. The six modules interact with each other, work together, and achieve integration.It can be seen from the integration relationship shown in Fig. 3 that the final result is the management of enterprise customer files, product files, other customer files, etc., as well as the standardization of basic data and user data of each department. At the same time, the main data specifications of the system also lay the foundation for the establishment of contract management and financial sharing platforms. In the contract management system, as the enterprise's ERP and financial sharing platform, the sales management with ERP is based on the order-based interaction of orders and contracts, while the financial sharing platform is the execution process of the contract, which analyzes the confirmation and execution of the contract's income, collection, and realizes two-way interaction.

(2) Realization of production-to-cost integration strategy

**Figure 4:**
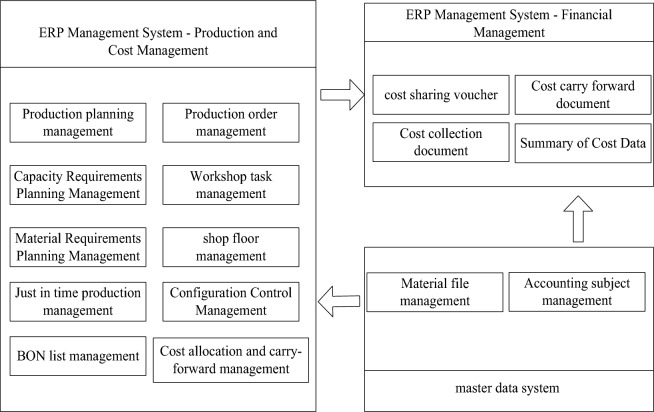
Production-to-cost integration strategy implementation integration diagram.

Combined with the “manufacturing-cost integration” strategic design, in terms of implementation, each functional module of ERP must be combined with the main data management system to form an integrated application from manufacturing to cost^25^. In the comprehensive application, it is necessary to give full play to the functional advantages of each system, and do a good job in the integration management between the various systems to achieve a better display of the effect after implementation, as shown in Fig. 4:

The production and cost management in the ERP management system in Fig. 4 consists of 10 modules. The production and cost management results obtained from the system are transmitted to the financial management module of the ERP management system. At the same time, the data in the main data system is transmitted to the production and cost management module and the financial management module. The financial management module is responsible for the final financial analysis. It can be seen from the integration relationship shown in Fig. 4 that the final result is that the master data system provides a standardized data basis for the production management module in the ERP system, and standardizes the basic data of each unit. ERP system refers to the front-end management of an enterprise. It integrates its own production and operation functions and financial management functions to form cost allocation vouchers, cost carry-over vouchers, cost collection vouchers, and a summary of cost information.

(3) Realization of other business integration strategies

**Figure 5:**
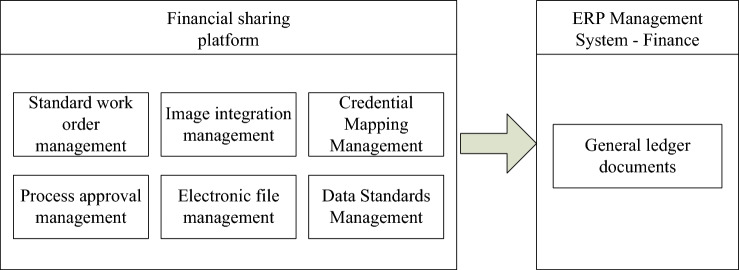
Other business integration strategy implementation integration diagram.

In terms of other business integration, the group must unify the functions of the financial sharing platform and ERP system and integrate it with other businesses. In the comprehensive application, it is necessary to give full play to the functional advantages of each system, and do a good job in the integration management of each system, so as to achieve a better display effect after implementation, as shown in Fig. 5.

The financial sharing platform in Fig. 5 includes six modules: standard work order management, image integration management, credential mapping management, process approval management, electronic file management, and data standard management. The finance module of the ERP management system is responsible for transmitting the data from these six modules to the finance module and generating the general ledger file. From the integration relationship shown in Fig. 5, it can be seen that the final implementation effect of the system is to use the financial sharing platform to design other types of business, and to use its process approval management, document mapping management and other functions so as to manage other businesses. Through the interface and the financial module of the ERP system, the system is integrated, so that the voucher data is automatically transmitted, and the financial data of the ERP system is more complete^26^. In financial management, image integration and electronic file management system are used to make file management electronic and visual, establish accounting electronic files, and lay the foundation for remote management of enterprises.

### Financial management and risk warning based on intelligent algorithms

In the past few years, overseas scholars have established many prediction models and methods. In recent years, many scholars have introduced artificial intelligence technology in financial risk prediction and improved it. Back propagation neural network is a dynamic model produced by imitating the nervous system of the brain^27^.

1. Back propagation (BP) neural network analysis

**Figure 6:**
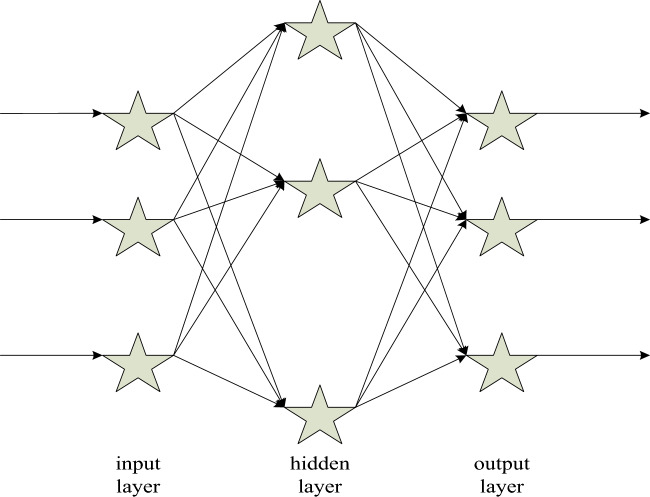
Schematic diagram of BP neural network structure.

The famous “error backpropagation” neural network was proposed in 1985, which is a forward-forward neural network with one or more layers^28^. It not only has the characteristics of parallel processing, distributed storage, fault tolerance, etc., but also has the characteristics of self-learning, self-organization, and adaptability^29,30^. The BP algorithm does not need to make strict assumptions about the input variables, but just introduces noise into the input variables, with no need to know the relationship between variables and factors, as shown in Fig. 6.

From Fig. 6, it can be inferred that the algorithm is as follows. The input variables of the BP neural network are set as Formula 1:

1 $$X = (x_{1} ,x_{2} ,K,x_{n} )^{T}$$

Then the corresponding output variable is as Formula 2:

2 $$Y = (y_{1} ,y_{2} ,K,y_{m} )^{T}$$

The input of each neuron in the hidden layer is set to Formula 3:

3 $$b_{j} = \sum\limits_{i = 1}^{n} {W_{ij} x_{i} - \theta_{j} }$$

In Formula 3, $j = 1,2,\Lambda ,k,W_{ij}$ is the connection weight from the input layer to the hidden layer. $\theta_{j}$ is the threshold of the hidden layer neuron *y*. *k* is the number of hidden layer units. If the activation function adopts a sigmoid function:

4 $$f(x) = \frac{1}{{1 + e^{ - x} }}$$

then the output of the hidden layer unit is as Formula 5:

5 $$S_{j} = \frac{1}{{1 + \exp \left( { - \sum\limits_{i = 1}^{n} {W_{ij} x_{i} + \theta_{j} } } \right)}}$$

The input of each output layer neuron is as Formula 6:

6 $$C_{t} = \sum\limits_{j = 1}^{q} {V_{ij} S_{j} - \lambda_{t} }$$

The output of each output layer neuron is as Formula 7:

7 $$L_{t} = \frac{1}{{1 + \exp \left( { - \sum\limits_{j = 1}^{q} {V_{jt} S_{j} + \lambda_{t} } } \right)}}$$

In Formulas 6 and 7, $t = 1,2,\Lambda ,q,V_{jt}$ is the connection weight from the hidden layer to the output layer. *t* is the output layer unit threshold. In the process of error backpropagation, the single sample error $E_{k}$ is as Formula 8:

8 $$E_{k} = \frac{{\sum\limits_{t = 1}^{p} {(y_{t}^{k} - L_{t}^{k} )^{2} } }}{2}$$

The total system error *E* is as Formula 9:

9 $$E = \sum\limits_{k = 1}^{m} {E_{k} }$$

If both $E_{k}$ and *E* are less than the allowable error, the training process ends; otherwise, the output deviation of each layer of nodes is calculated, and the network connection weights and thresholds are modified. In order to keep decreasing according to the gradient principle, then there is Formula 10:

10 $$\Delta V_{jt} = - \alpha \partial E_{k} /\partial V_{jt}$$

From Formula 10, it can be deduced that the weight adjustment of the output layer is as Formula 11:

11 $$\Delta V_{jt} = ad_{t}^{k} b_{j}$$

In Formula 11, $d_{t}^{k}$ is as Formula 12:

12 $$d_{t}^{k} = (y_{j}^{k} - L_{j}^{k} )t(1 - L_{t}^{k} )$$

$\alpha$ is the learning rate, and the output layer threshold adjustment amount is as Formula 13:

13 $$\Delta \lambda = \alpha d_{t}^{k}$$

Similarly, the weight adjustment of the hidden layer is as Formula 14:

14 $$\Delta W_{ij} = \beta e_{j}^{k} x_{i}$$

The threshold adjustment amount of the hidden layer is as Formula 15:

15 $$\Delta \theta_{j} = \beta e_{j}^{k}$$

In Formulas 14 and 15, there are $i = 1,2,\Lambda ,q$, $j = 1,2,\Lambda ,{\text{p}}$ and $k = 1,2,\Lambda ,m$. Therefore, there is Formula 16:

16 $$e_{j}^{k} = \left( {\sum\limits_{t = 1}^{q} {d_{t}^{k} v_{jt} } } \right)S_{j} (1 - S_{j} )$$

The weights and thresholds of the output layer and the hidden layer are adjusted according to the derived adjustment amount, so that the entire model completes one training.

The specific implementation process of BP neural network in financial management and risk warning is as follows:

Firstly, collect a large amount of financial management and risk warning related data, and preprocess the data, including data cleaning, standardization, etc. Then, based on the actual problem requirements, determine the structure of the BP neural network, including the number of nodes in the input layer, output layer, and hidden layer. Afterwards, network training is carried out, using sample data to train the network. The weights and thresholds of the network are continuously adjusted through backpropagation algorithms to reduce errors. Finally, predict and evaluate the new data using the trained network, and evaluate the effectiveness of financial management and risk warning based on the prediction results.

(2) Analysis of particle swarm optimization (PSO) algorithm

Particle swarm optimization is similar to other evolutionary algorithms in which it also uses the concepts of “swarm” and “evolution”. Compared with other evolutionary algorithms, the PSO algorithm adopts evolutionary operation^31,32^.

If the current position of particle i is $X_{i}$, that is, the current flying speed of particle i is $V_{i}$, then the best position passed by particle i is $P_{i}$, that is, the best fitness value experienced by particle $i_{1}$ is called the individual best position. For the convenience of discussion, assuming that $f(x)$ is the smallest objective function, then the current best position of particle i is determined by Formula 17:

17 $$P_{i} (t + 1) = \left\{ {\begin{array}{*{20}c} {P_{i} (t),f(X_{i} (t + 1)) \ge f(P_{i} (t))} \\ {X_{i} (t + 1),f(X_{i} (t + 1)) < f(P_{i} (t))} \\ \end{array} } \right.$$

Assuming that the number of particles in the group is S, and the best position experienced by all particles in the group is $P_{g} (t)$, which is called the global best position, then there is Formula 18:

18 $$P_{g} (t) \in \left\{ {P_{0} (t),P_{1} (t),\Lambda ,P_{s} (t)} \right\}\left| {f(P_{g} (t))} \right.$$

With the above definitions, the evolution formulas of the basic particle swarm algorithm can be described as Formula 19 and Formula 20:

19 $$vi_{j} (t + 1) = v_{ij} (t) + c_{1} r_{1j} (t)[p_{ij} (t) - x_{ij} (t)] + c_{2} r_{2j} (t)[p_{gj} (t) - x_{ij} (t)]$$

20 $$x_{ij} (t + 1) = x_{ij} (t) + v_{ij} (t + 1)$$

BP neural network has broad application prospects in a wide range of application fields, but its drawbacks are becoming more and more obvious, which seriously restricts the applicability of BP network. Many scholars have made certain improvements to the BP algorithm, but the results obtained are not the same. At present, the most popular method is to combine the BP algorithm with the neural network. It is structurally optimized due to improvements in connection weights, thresholds, etc. Biological intelligence algorithm is one of the most widely used algorithms, such as genetic algorithm, particle swarm algorithm and so on. The local minimization problem of BP network is carried out by genetic algorithm, but it has a large amount of calculation, many network parameters and poor operability, while PSO has better global optimization ability, and on the basis of existing theory, it has better BP network optimization effect.

The specific implementation process of particle swarm optimization algorithm in financial management and risk warning is as follows:

In the implementation process of particle swarm optimization algorithm in financial management and risk warning, firstly, it is necessary to initialize the particle swarm and randomly generate a set of solutions, each corresponding to a parameter or strategy of the financial management and risk warning model. Afterwards, based on the actual needs of the problem, design a fitness function to evaluate the superiority or inferiority of each particle. In financial management and risk warning, fitness functions can be designed based on prediction errors, risk indicators, etc. Next, according to the rules of particle swarm optimization algorithm, update the position and velocity of particles to search for better solutions. In financial management and risk warning, the optimal solution is found by iteratively updating the parameters or strategies of particles. Subsequently, the termination condition is determined to determine whether the algorithm has reached the termination condition, such as reaching the preset maximum number of iterations or finding the optimal solution that meets the requirements. Can we obtain the optimal solution again and apply it to practical financial management and risk warning to improve decision-making effectiveness and risk management level.

### Challenges

Because artificial intelligence and big data have inherent biases and limitations, if the data comes from past unfair social structures or decisions, then these data may contain these biases. Machine learning models, especially supervised learning models, inherit biases from their training data. Secondly, due to limitations in data collection, such as web crawlers only being able to crawl information from public web pages, or users being unwilling to share certain sensitive information, the collected data is not comprehensive. In addition, big data also has issues with information accuracy. When the model is too complex or the training data is too sufficient, the model may remember the noise in the training data instead of learning the basic rules of the data. Finally, the interpretability of machine learning models is a challenge. For some complex models, such as deep neural networks, the internal operating mechanisms are difficult to understand intuitively. That is to say, although these models perform well in certain tasks, their decision-making process is “black box”, and it is difficult for people to know why the models make a certain decision.

When dealing with financial datasets, model constraints and potential overfitting issues are particularly important. Financial datasets typically have characteristics such as large data volume, high data dimensionality, non-linear relationships, temporal correlation, high noise, and anomalies. Due to market fluctuations and data collection issues, there may be a large amount of noise and outliers in financial data. These characteristics pose challenges to the constraints and overfitting issues of the model. Due to the high dimensionality and non-linear relationships of financial data, models need to have a certain level of complexity to capture these features. However, excessive complexity may also lead to a decrease in the generalization ability of the model, that is, overfitting the training data and a decrease in the predictive ability of unknown data. Overfitting and underfitting: When training a model, if the model is too complex, it may overfit the training data, resulting in poor performance on new and unseen data. On the contrary, if the model is too simple, it may not be able to capture the complex patterns of the data, resulting in underfitting. In the financial field, overfitting may lead to models fitting past data well, but having poor predictive ability for the future. When dealing with financial datasets, special attention should be paid to the constraints and overfitting issues of the model. By reasonable model selection, preprocessing, feature engineering, and validation, the performance of the model can be maximized and potential risks can be reduced.

### Informed consent

All authors have read and agreed to the published version of the manuscript.

## Financial management and control experiment based on bp neural network

### Selection of indicators affecting financial risk and establishment of indicators

The selection criteria for financial risk indicators in this article should include comprehensiveness, sensitivity, operability, stability, and comparability. The selection of indicators should cover all aspects of the enterprise, including financial status, operational performance, debt paying ability, profitability, and growth ability. The selected indicators should be able to sensitively reflect changes in the financial status of the enterprise. The selection of indicators should consider their operability in practice. This includes the availability of data, ease of calculation, and stability of indicators. At the same time, indicators should have a certain degree of stability to avoid excessive fluctuations caused by external environment or specific factors of the enterprise. In addition, the selected indicators should be comparable in order to be compared at different times or between different enterprises. This helps to assess the financial risks of enterprises in a larger context and provides a basis for comparison between enterprises.

ZH Group's financial risk index reflects the operation status of the enterprise from all aspects of the enterprise. The analysis was carried out from five aspects: the long-term solvency, short-term solvency, operation ability, profitability and development ability of the enterprise, as shown in Table 1.

**Table 1:** Financial risk indicators.

Category	Numbering	Indicator name
Short-term solvency	X1	Current ratio
	X2	Quick ratio
	X3	Cash ratio
Long-term solvency	X4	Assets and liabilities
	X5	Equity to Debt Ratio
Operational capability	X6	Inventory turnover
	X7	Accounts Receivable Turnover
	X8	Total asset turnover
Profitability	X9	Return on assets
	X10	Operating profit margin
	X11	Cost profit margin
Growth ability	X12	Indicator name
	X13	Net profit growth rate
	X14	Operating profit growth rate

It can be seen from Table 1 that after the five indicators of the short-term solvency, long-term solvency, long-term solvency, operation capacity, profitability, and growth of the enterprise have been preliminarily screened, the financial risks of ZH Group were finally determined, including 3 short-term solvency, 2 long-term solvency, 3 profitability, and 3 growth capability indices. A total of 14 core indicators were selected.

The indicators such as current ratio, quick ratio, and bank cash reserve ratio are very important for the group company, as they directly affect the short-term solvency and liquidity of the enterprise. Maintaining sufficient liquidity and cash flow is crucial for the survival and development of enterprises, as it can ensure their normal operation and payment ability. Indicators such as asset to liability and equity to debt ratio are used to evaluate a company’s debt level and capital structure. For group companies, reasonable control of debt levels and optimization of capital structure can reduce financial risks and ensure the stable development of the enterprise. Indicators such as inventory turnover, accounts receivable turnover, and total asset turnover can help group companies evaluate their operational efficiency and profitability. Indicators such as return on assets, operating profit margin, and cost profit margin provide a basis for decision-making analysis for the group company, helping the enterprise understand its own profitability and operational efficiency. Meanwhile, indicators such as net profit growth rate and operating profit growth rate can help the group company formulate strategic planning, clarify the development direction and goals of the enterprise. By comprehensively analyzing various financial indicators, the group company can better evaluate its own financial risks and take effective measures to control them.

Based on the analysis of these indicators, 14 indicators related to financial risks were selected. These indicators were strongly correlated with each other, and a large number of information duplications might inevitably occur, thereby greatly increasing the difficulty of analyzing and solving problems. Through principal component analysis, the linear correlation variables of the financial index can be transformed into several independent principal components, thus reducing the computational complexity of the neural network.

BP neural network is a multi-layer feedforward network trained through backpropagation algorithm. It can learn and store a large number of input–output mapping relationships without the need to know the mathematical equations between inputs and outputs beforehand. When using BP neural network for financial analysis, the above financial indicators can be selected as input data. The reason is that these financial indicators provide rich data features that are suitable for input into BP neural networks for financial risk analysis. Neural networks can automatically learn and simulate complex relationships between financial data, improving the accuracy of prediction and classification. Through the analysis of neural networks, enterprises can gain in-depth insights into the sources of financial risks and take effective measures to control them. In addition, the interpretability of BP neural networks makes the analysis results more practical, helping companies understand their financial risks and make targeted improvements.Therefore, this paper firstly normalized the data of financial risk index. Then, using the International Business Machines Corporation (IBM) SPSS (Statistical Product Service Solutions) statistical software to conduct principal component analysis on 14 financial risk indices, the results are shown in Table 2:

**Table 2:** Common factor variance.

	Initial	Extraction
× 1	1.000	0.966
× 2	1.000	0.975
× 3	1.000	0.898
× 4	1.000	0.752
× 5	1.000	0.951
× 6	1.000	0.909
× 7	1.000	0.896
× 8	1.000	0.544
× 9	1.000	0.832
× 10	1.000	0.776
× 11	1.000	0.846
× 12	1.000	0.457
× 13	1.000	0.964
× 14	1.000	0.962

As can be seen from Table 2, among the 14 financial risk indices, the common factor variance represents the ratio of information extracted from each initial variable by the principal component analysis. The overall changes in each indicator are shown in Table 3:

**Table 3:** Interpretation of total variance.

Component	Initial eigenvalues			Extraction sums of squared loadings		
	Total	% of variance	Cumulative%	Total	% of variance	Cumulative%
1	3.896	27.823	27.821	3.894	27.821	27.828
2	2.588	18.493	46.315	2.586	18.496	46.315
3	1.787	12.767	59.086	1.786	12.764	59.083
4	1.416	10.095	69.184	1.412	10.095	69.183
5	1.022	7.342	76.524	1.023	7.346	76.526
6	1.002	7.142	83.676	1.000	7.144	83.675
7	0.791	5.689	89.353			
8	0.763	5.447	94.797			
9	0.341	2.483	97.286			
10	0.131	0.934	98.214			
11	0.106	0.776	98.992			
12	0.070	0.505	99.494			
13	0.049	0.342	99.835			
14	0.024	0.162	100.000			

It can be seen from Table 3 that the total variance explained by all the main components represents the explanation of the total variance of the original variable by each principal component. In this SPSS software, the default setting is Eigenvalues was 1, so only 6 principal components were retained, which contained 83.675% of the information.

### Prediction of financial risk by BP model

PSO-BP neural network is a new financial risk prediction method. Its main idea is to optimize the initial weight and threshold of the network through the PSO algorithm, and use it as a new weight and threshold^33^.

1. Training process of the PSO-BP model

Using MATLAB-R2014a to simulate the BP model, the results showed that the method was effective. In the above, the dimensionality reduction of each index at the input end of the model was carried out, and 6 main variables were obtained, and 6 parameters were used as input data. The model adopted one-dimensional risk assessment, and the number of neurons in the output layer of the PSO-BP model was determined to be 1. Confirming the number of neurons in the middle hidden layer was more complicated. Because it is related to the number of input and output nodes, there is no effective selection method so far. It is only based on empirical formulas and combined with experiments to find the optimal number of hidden layer nodes. The experimental results showed that when the number of 10 hidden layer nodes was 10, the network reached a state of convergence and had good convergence. Therefore, the improved BP neural network was simulated in this paper, and its structure was 6-10-1.

**Figure 7:**
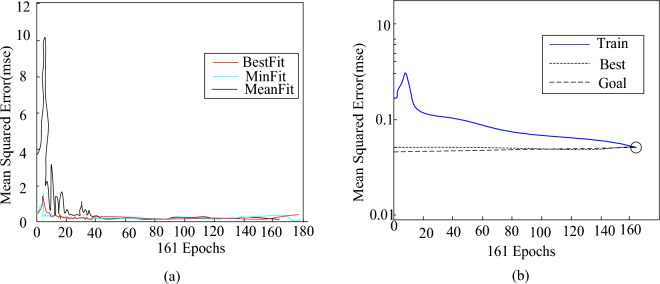
PSO-BP network training effect diagram. (**a**) Optimal individual fitness of PSO-BP algorithm training. (**b**) Change of mean square error during PSO-BP network training process.

Based on the above model boundary settings, 150 examples of the example set were utilized for the learning of the PSO-BP model, and 50 examples were used to predict the model that met the accuracy requirements, in order to test the correctness of the model. The operation results of PSO calculation were limited to track the maximum number of cycles or reach the expected error, and the fitness function of PSO was the mean square error function of the calculation error of BP neural tissue. BP’s “LM algorithm” is a method of learning and training, as shown in Fig. 7:

As shown in Fig. 7a, the adaptation value of the PSO algorithm changed significantly during the iteration, and the error was gradually reduced. After 161 iterations, the variance dropped to 0.1518. As shown in Fig. 7b, the BP model was optimized by principal component analysis and PSO method. After 161 times of training, the overall error of the model was kept within 5%, and it can effectively overcome the shortcomings of the traditional BP model.

(2) Analysis and comparison of model effects

After the training of the PSO-BP network, the empirical analysis was carried out using the experimental samples. Through the experimental comparison between the BP neural network and the genetic algorithm-back-propagation neural network (GA-BP neural network), it was found that the differences in the prediction accuracy of the BP model, the GA-BP model and the PSO-BP model were compared in Table 4.

**Table 4:** Comparison of prediction results of BP model, GA-BP model and PSO-BP model.

	Number of test samples	Number of misjudgments	Correct rate/%
BP prediction model	50	17	67.0
GA-BP prediction model	50	12	77.0
PS0-BP prediction model	50	7	85.0

It can be seen from Table 4 that the prediction accuracy of PSO-BP reached 85.0%, which was a significant improvement over the traditional BP model of 67.0% and the GA-BP model of 77.0%. The results showed that the PSO-BP model had a good application prospect in predicting financial risks. In practical application, the BP neural network was modified by principal component analysis method and PSO method, thus providing a better reference for enterprises and investors. In order to improve the prediction accuracy, a BP neural network optimization model based on PSO optimization was established by using the PSO method and the BP model. On this basis, the PSO method was used to quickly search for the optimal overall optimal, and the method was used in the financial risk prediction of ZH Group, and compared with the BP model and the GA-BP model.

In addition to the accuracy testing of the above model, in order to better verify the accuracy of the model in financial forecasting, the average accuracy and standard deviation of financial forecasting before and after using the model were also calculated. The results are shown in Table 5:

**Table 5:** Comparison of financial forecast accuracy before and after use.

Year	Before use	After use	*p*-value
2015	72.51 ± 4.32	89.41 ± 3.19	0.013
2016	70.42 ± 3.95	86.95 ± 6.19	0.024
2017	71.33 ± 3.14	88.57 ± 5.36	0.018
2018	73.14 ± 5.18	89.16 ± 3.49	0.009
2019	72.62 ± 3.15	86.43 ± 7.11	0.012

As shown in Table 5, for the financial forecasts of enterprises from 2015 to 2019, the accuracy of the model used in this article is significantly higher than before, with a *p*-value of *p* < 0.05, and the difference is statistically significant. This indicates that the model proposed in this article can significantly improve the accuracy of financial forecasting, and computer intelligent algorithms have good application value in financial forecasting.

### Intelligent optimization of financial management and control of ZH group

1. Analysis of group management and control effect

The financial management of ZH Group can affect the operation of ZH Group to a large extent. Through the collection and arrangement of ZH Group's financial data from 2015 to 2019, this paper conducted a horizontal analysis of ZH Group's operating capabilities, which can reflect the weakening trend of ZH Group's control ability from the side. This paper also collected and organized the financial data of the two major groups B and C from 2015 to 2019, and analyzed the company's operating conditions from a horizontal perspective, which found the differences between the company and its peers, providing guidance for subsequent research and recommends the best options for financial control.

**Figure 8:**
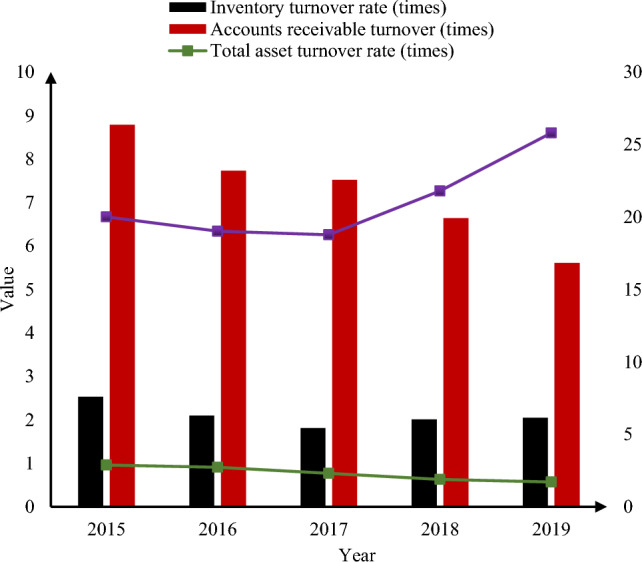
Analysis of ZH Group’s operational capability from 2015 to 2019.

Operational capability is the effective use of resources by an enterprise. From a practical point of view, because it reflects the efficiency of the financial management of the enterprise, the operational capabilities of the ZH Group were analyzed. From 2015 to 2019, the operation capability analysis of ZH Group is shown in Fig. 8:

As shown in Fig. 8, the accounts receivable of ZH Group decreased by 3.18 times in 5 years, which indicated that the company's accounts receivable recovery efficiency was very low, and the profitability of funds was also affected by the unreached accounts, which were very detrimental to the sustainable development of the company. Therefore, in order to carry out reasonable financial control, it is necessary to do the corresponding accounts receivable management to prevent huge losses in operation. The turnover rate of fixed assets increased by 5.79 times, and the change was not large, indicating that the projects that ZH Group had bid for in the past 5 years were relatively reasonable, and the utilization rate of fixed assets was also high, which was beneficial to the long-term development of the company. In addition, the inventory turnover rate dropped by 0.48 times in 5 years, indicating that the management of materials purchased and put into production by ZH Group was slightly lacking, and there might be inventory problems. The turnover rate of total assets decreased by 0.39 times, which indicated that the operating scale of ZH Group had changed in the past 5 years, and the overall operating level still needed to be improved.

**Figure 9:**
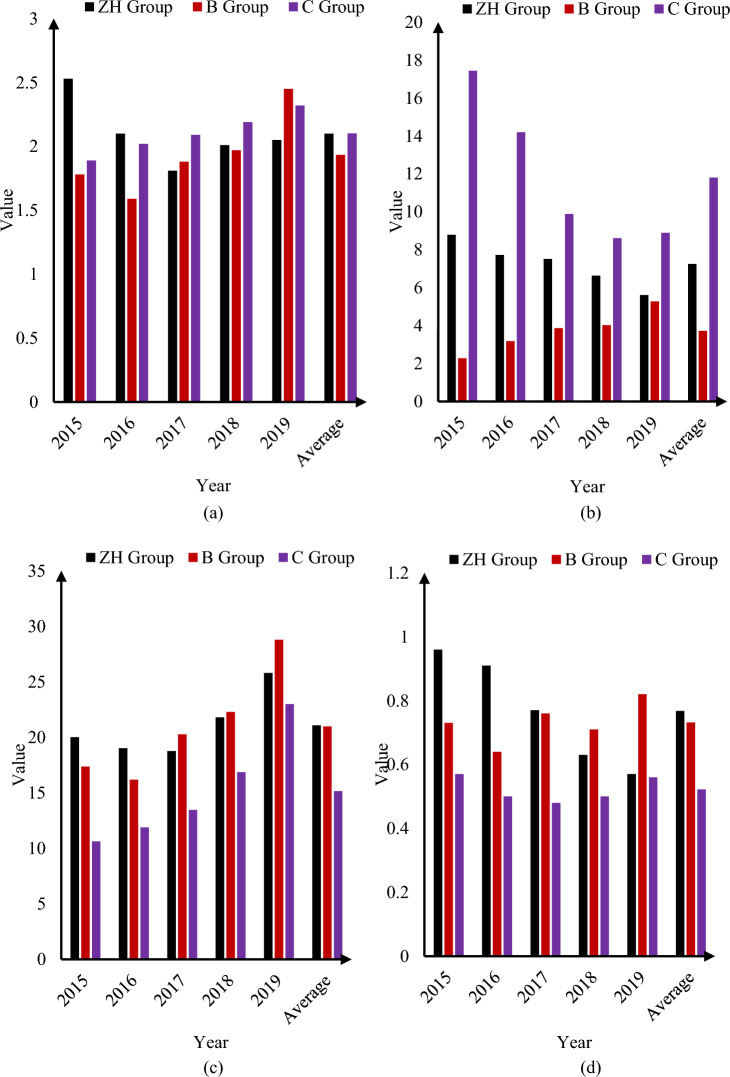
Comparative analysis of ZH, B, C Groups. (**a**) Inventory turnover ratio (times). (**b**) Accounts receivable turnover ratio (times). (**c**) Turnover rate of fixed assets (times). (**d**) Turnover rate of total assets (times).

This paper selected two companies, B and C, as the comparative research objects in order to better analyze the operational capabilities of the ZH Group and highlight the problems existing in the financial management of the ZH Group, as shown in Fig. 9:

As can be seen from Fig. 9a, the inventory turnover ratios of Groups B and C showed an overall trend of increasing year by year, and the inventory turnover of ZH Group first decreased and then increased. Inventory turnover in recent years has not reached the highest level in 5 years, and inventory management has also been lacking, which indicated that ZH had some problems in controlling the inventory. In the trend of accounts receivable turnover ratio in Fig. 9b, the trend of B Group and C Group was just opposite. The average annual accounts receivable turnover ratio of ZH Group in the three companies of ZH, B and C was at a medium level, but there was a large risk of non-performing loans, which indicated that there was a problem with ZH Group's money management. In particular, the use of funds was inefficient, and a large amount of funds were used in accounts receivable. In Fig. 9c, the fixed asset turnover ratios of the three major groups were all on the rise, with Group B taking the lead, while ZH Group's fixed asset turnover ratio was between B Group and C Group, but there was still room for growth. According to the operating characteristics of ZH Group, some of the company’s businesses were surveying and designing, and the value of the fixed assets used was relatively low, which showed that ZH Group had a higher fixed asset turnover rate due to its operating structure.

As shown in Fig. 9d, in the past 5 years, the total asset turnover rate of ZH Group had been on a downward trend, while in the last 3 years, the overall asset level of B Group had gradually decreased, and its industry advantage had also disappeared, indicating that ZH's overall operating ability was declining and its financial controls must be optimized. Judging from the above indicators, ZH Group’s operating capacity was in a state of gradual decline. Its business development was in general, and its competitive advantage was constantly weakening. The quality of an enterprise's operational capabilities not only reflects the operation and profitability of ZH Group, but also reflects ZH Group's use and control of capital.

**Figure 10:**
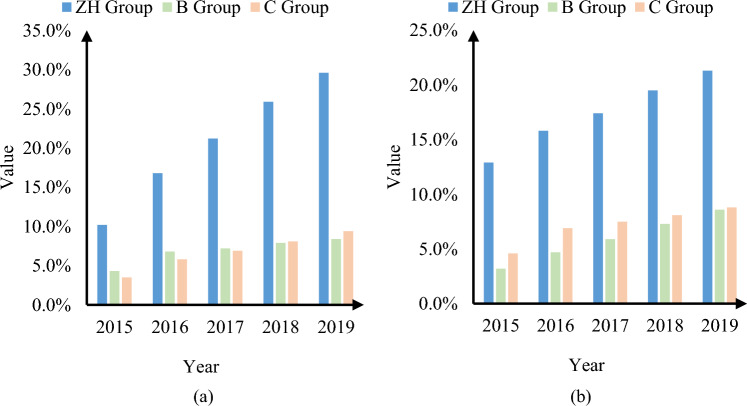
Operational improvements and financial performance improvement in different years. (**a**) Operational improvements in different years; (**b**) Financial performance improvement in different years.

In addition to the analysis of operational capabilities mentioned above, this article also provides statistics on operational improvement and financial performance improvement in different years. The result is shown in Fig. 10:

According to Fig. 10a,b, it can be seen that the ZH enterprise using the method proposed in this article improved its operations by 10.2% and financial performance by 12.9% in 2015; B Company, which used traditional methods, saw a 4.3% increase in operational improvement and a 3.2% increase in financial performance in 2015; C Company, which used traditional methods, saw a 3.5% increase in operational improvement and a 4.6% increase in financial performance in 2015. In 2019, the ZH enterprise using the method described in this article improved its operations by 29.6% and its financial performance by 21.3%; The operational improvement of Company B using traditional methods has increased by 8.4%, and financial performance has increased by 8.6%; The operational improvement of Company C using traditional methods increased by 9.4%, and financial performance improved by 8.8%,. From it, it can be seen that with the delay of time, the operational improvement and financial performance improvement of ZH enterprise using the method proposed in this article are more significant. This indicates that using the method proposed in this article is more conducive to achieving intelligent management of financial big data and better promoting the improvement of enterprise financial performance.

(2) ZH Group’s financial management and control optimization plan

The previous article has analyzed the problem of enterprise control mode, and believes that the current control mode of ZH Group is financial management, while the financial control of its group is power control. Three thoughts are put forward for how to optimize the management model of ZH Group:

First, this paper analyzes the choice of control mode from the aspects of the external market economic environment, the business characteristics and organizational structure of the enterprise, and the group’s overall development strategy. ZH Group is part of the construction industry. First, over the years, due to the increasingly fierce competition in engineering construction, with more and more engineering investment, the company’s profit margin is getting lower and lower, and ZH Group’s market is getting smaller and smaller. There must be more benefits from management.

Second, to determine the degree of centralized control over a subordinate enterprise, when considering the degree of centralized control, it is necessary to take into account its own business characteristics and management capabilities. Within the enterprise, the overall strategic development of the enterprise and the control ability of the enterprise should be considered, that is, the control resources of the enterprise are limited, and the key must be grasped. Therefore, subsidiaries must be divided into two categories: strategic core and non-strategic core according to their position in the group strategy. Centralized control is implemented for enterprises with strategic core business, and relatively decentralized control is implemented for enterprises without strategic core business^34^.

Third, it is necessary to weigh which powers are concentrated and which powers are decentralized for strategic overall planning and resource allocation at the group level, giving subsidiaries complete autonomy, and delegating power to subordinates. Meanwhile, it is necessary to precisely control the delegation and degree of authority according to the operation status of the subsidiary and the strategic intention of the group.

To apply the financial control solution designed in this article to small organizations, the key is to use intelligent algorithms to effectively manage limited financial data, in order to reduce costs and improve efficiency. For large enterprises, it is necessary to design more complex systems in the financial control strategy of this article, combined with big data and machine learning algorithms, for financial risk warning and strategic decision support. Regardless of scale, the key is to ensure data quality and accuracy. Therefore, for any organization, when implementing financial control solutions, attention should be paid to data cleaning, integration, and validation to ensure that intelligent algorithms can provide accurate and reliable financial insights. Meanwhile, continuous technological updates and personnel training are also necessary to adapt to constantly changing business needs and market environments.

Computer intelligent algorithms have played a core role in the field of financial big data management, bringing breakthrough development to this field. Traditional financial theories are often based on linear models and historical data, while computer intelligence algorithms can reveal nonlinear relationships and potential patterns, providing more accurate basis for financial decision-making. Computer intelligent algorithms have injected new vitality into the field of financial big data management. Through deep mining and analysis of massive data, we can gain a deeper understanding of market dynamics, risk assessment, and investment strategies. This not only challenges traditional theories, but also prompts scholars and industry professionals to rethink the essence and laws of finance. Therefore, financial big data management and intelligence based on computer intelligence algorithms are important driving forces for theoretical discussions in the financial field, laying a solid foundation for future research and application.

Overall, computer intelligence algorithms provide powerful analytical tools for financial big data management. Through advanced algorithms, we can more accurately mine the patterns behind the data, thereby providing strong support for investment decisions. At the same time, combined with traditional financial theory, these intelligent algorithms provide a new perspective, enabling us to have a more comprehensive understanding of the market's operational mechanisms. More importantly, financial big data analysis based on computer intelligence algorithms does not fully conform to traditional financial models. This not only challenges our traditional understanding of the market, but also brings many new ideas to the financial field. For example, through big data analysis, we found that certain nonlinear relationships play an important role in the market, which led us to re-examine traditional linear models. Overall, the combination of computer intelligence algorithms and financial big data provides us with deeper and more comprehensive market insights. This not only helps us make wiser investment decisions, but also provides strong support for the improvement of financial theory and models.

However, in research, attention should also be paid to the importance of the integrity of financial big data in the analysis and decision-making process driven by artificial intelligence. High quality data is the foundation for ensuring the accuracy of results. If there is bias or poor quality in the data, artificial intelligence algorithms may produce misleading conclusions. For example, if the dataset ignores certain important market dynamics or only contains data for specific groups, models trained based on these data will not be able to fully reflect market conditions. Therefore, when making decisions using financial big data, it is necessary to pay attention to the diversity and completeness of data sources, and appropriately clean and verify the data to ensure its quality and accuracy. This can not only improve the accuracy of analysis, but also avoid decision-making errors caused by data quality issues.

## Conclusions

With the advancement of computer technology, the application of intelligent algorithms is becoming increasingly widespread. This article explores the financial control of large groups from two perspectives: the needs of financial management and the implementation of information systems.Based on the actual situation of ZH Group, this paper has carried out financial control on other large-scale group enterprises. The article introduces the operation status of ZH Company, and points out the need of the company's financial control in combination with the current technology and management development trend. Combined with the actual situation of the company and other projects, the overall design and implementation effect of the corporate financial control strategy is given. In addition, this article combines particle swarm optimization algorithm and BP neural network to construct the latest financial prediction model, and through comparison with other traditional models, it is found that this model has better prediction accuracy. Research has shown that the use of computer intelligence algorithms can significantly improve the accuracy of financial risk prediction, enable enterprises to better cope with risks, and thus better achieve the intelligence of financial big data management. This study demonstrates the important significance of computer intelligence algorithms in the field of financial management and their good practical value. However, due to the limitations of theory and practice, the breadth and breadth of the research cannot be accurately estimated, and many problems cannot be well explained, which need to be discussed in detail in combination with the actual situation. The data used in the study may only cover part of the financial information of Zhonghong Group, which may limit the comprehensive evaluation of its operational capabilities. When using computer intelligence algorithms for data analysis, the choice of models and methods may directly affect the accuracy and effectiveness of research results. Subsequent research needs to comprehensively evaluate the operational capabilities of Zhonghong Group by adding more financial and non-financial data (such as market trends, competitor information, etc.). In addition, in the financial field, data privacy is a core issue. With the widespread application of big data and intelligent algorithms, it has become crucial to ensure the security and privacy of data. Future research should delve into how to protect personal privacy when collecting, storing, and using financial data, while ensuring the legality and security of the data. The use of intelligent algorithms for financial decision-making may bring a series of ethical issues. Future research should focus on discussing ethical principles and responsibilities in financial big data management and intelligent applications, and seek to establish corresponding ethical norms and guidelines. With the development of financial technology, regulatory agencies need to face new challenges, such as how to ensure data compliance, how to regulate algorithmic decision-making, and how to protect consumer rights. Future research should focus on the challenges faced by regulatory agencies in implementing such intelligent systems, as well as how to formulate appropriate policies and regulations to promote the healthy development of the financial industry. For large-scale deployment of intelligent systems, many technical and organizational challenges need to be addressed. Future research should delve deeper into these challenges and propose innovative solutions.

## Data Availability

Datasets generated and/or analyzed during the current study are available from the corresponding author on request.
